# The *Leptospira* immunoglobulin-like protein LigB from *Leptospira borgpetersenii* serovar Arborea is not required for either acute or chronic infection

**DOI:** 10.1128/iai.00662-25

**Published:** 2026-03-19

**Authors:** Luis Guilherme Virgilio Fernandes, Ellie J. Putz, Jarlath E. Nally

**Affiliations:** 1 Infectious Bacterial Disease Research Unit, USDA Agricultural Research Service, National Animal Disease Center 57837 , Ames, Iowa, USA; St Jude Children's Research Hospital, Memphis, Tennessee, USA

**Keywords:** *Leptospira borgpetersenii*, LigB, virulence, mutagenesis, CRISPR-Prime Editing

## Abstract

Leptospirosis is a globally important zoonotic disease caused by more than 40 pathogenic *Leptospira* spp. that are responsible for more than 1 million human cases and almost 60,000 deaths annually. The disease also affects many companion, domestic, and wild animal species. Leptospiral immunoglobulin-like proteins (Lig), particularly LigA and LigB, are well-established surface membrane proteins that have been extensively studied due to their interactions with the host immune system. Silencing expression of both *ligA* and *ligB* in *L. interrogans* serovar Copenhageni results in attenuation of virulence, confirming their role as virulence factors. To examine the role of Lig proteins in other pathogenic species of *Leptospira*, we applied the CRISPR-Prime Editing (CRISPR-PE) strategy to engineer a one-nucleotide frameshift deletion in *ligB*, generating a knockout mutant in *L. borgpetersenii* serogroup Ballum serovar Arborea strain LR131, a pathogenic species that lacks *ligA*. Despite a complete loss of LigB expression, the mutant strain maintained its ability to cause acute lethal infection in hamsters and the ability to establish renal colonization in rats. These findings demonstrate that *ligB* alone is dispensable for both acute and chronic infection in the *L. borgpetersenii* strain LR131 background. This work represents the first targeted disruption of *ligB* in *L. borgpetersenii* as facilitated by CRISPR-PE and prompts a reevaluation of LigB as a universal virulence determinant in different genetic backgrounds. These insights are critical for advancing our understanding of leptospiral pathogenesis and guiding the design of broadly protective subunit vaccines.

## INTRODUCTION

Leptospirosis is a neglected zoonosis caused by pathogenic bacteria of the genus *Leptospira*, resulting in over 1 million human cases worldwide and almost 60,000 deaths annually (1, 2). Hosts can be infected after direct contact with leptospires present in biological fluids or indirectly by contact with contaminated moist environments (3, 4). To date, 43 pathogenic species are recognized, with hundreds of serovars, which are distinguished based on chemical and structural diversity of surface-exposed lipopolysaccharides (3, 5). The diversity of the genus is reflected in the genome composition of different pathogenic species, e.g., *L. interrogans* has a genome ~700 kb bigger than some *L. borgpetersenii* (6).

Though animal leptospirosis can result in significant morbidity and mortality in domestic and wildlife animals, they also act as reservoir hosts of infection that can shed *Leptospira* via urine for months to years. Rats are a reservoir host for *L. interrogans* serogroup Icterohaemorrhagiae serovar Copenhageni, the leading cause of leptospirosis in humans (1, 7). A recent epidemiology study conducted in the U.S. Virgin Islands (USVI) demonstrated that rats also act as a reservoir host for *L. borgpetersenii* serogroup Ballum, which has a genome size of approximately 4 Mb (8, 9). In contrast, mongooses in the USVI were reservoir hosts for *L. borgpetersenii* serogroup Sejroe (10). One of the rodent isolates, strain LR131, was further characterized as *L. borgpetersenii* serogroup Ballum serovar Arborea, causing an acute lethal infection in the hamster model of leptospirosis (8, 9).

CRISPR-based genetic tools have, for the first time, enabled the creation of site-directed targeted mutations in multiple pathogenic species and serovars of *Leptospira* (11–15). Most recently, the application of CRISPR-Prime Editing (CRISPR-PE) to *L. borgpetersenii* (14) demonstrates that functional genomics can now be performed in species other than *L. interrogans*. The ability to interrogate the role of specific virulence factors in different genetic backgrounds is an essential step toward understanding pathogenic mechanisms of infection.

Leptospiral immunoglobulin-like proteins (LigA, LigB, and LigC) are present in multiple pathogenic species of *Leptospira*; they play key roles in pathogenesis and interact with a myriad of host extracellular matrix and plasma components to mediate adhesion, inhibit hemostasis, and inactivate complement regulators (extensively reviewed by Haake and Matsunaga [16, 17]). Gene knockout of *ligB* by allelic exchange in *L. interrogans* serovar Copenhageni strain L1-130 did not affect virulence, which was hypothesized to be a result of functional redundancy from the presence of *ligA*, but not *ligC*, which presented as a pseudogene in this strain (18). Gene knockdown of both *ligA* and *ligB* by transcription activator-like effectors in *L. interrogans* serovar Manilae strain L495 resulted in attenuation of virulence despite the presence of an intact *ligC* (19). Similarly, gene silencing of both *ligA* and *ligB* in strain L1-130 using CRISPRi resulted in attenuation of virulence (20), further confirming the role of *ligA* and *ligB* as virulence factors.

The *ligB* gene appears to be distributed ubiquitously among all pathogenic strains, in contrast to *ligA* and *ligC* (21). Since *ligA* is absent in *L. borgpetersenii*, we generated a knockout mutant for *ligB* using CRISPR-PE to determine whether virulence was affected in the hamster model of acute leptospirosis or the rat model of renal colonization and excretion.

## RESULTS

### Construction and validation of the *ligB* knockout mutant in *L. borgpetersenii*

Plasmids pMaOriPE and pMaOriPEgRNAligB were sequenced to confirm their integrity (GenBank accession numbers PX766220 and PX766221, respectively). Unexpectedly, a small Cas9 fragment after the Cas9n-RT cassette was identified (Fig. S1) and is likely a carryover product from cloning during the construction of the original pMaOriPE (14). Since this sequence was present in all constructs, including those previously used to generate *lipL32* mutants in several strains of *Leptospira* (14), it is assumed not to interfere with the functionality of CRISPR-PE. Additionally, we confirm the presence of the PEgRNA targeting *ligB* for one-nucleotide deletion (Table S1).

**Fig 1:**
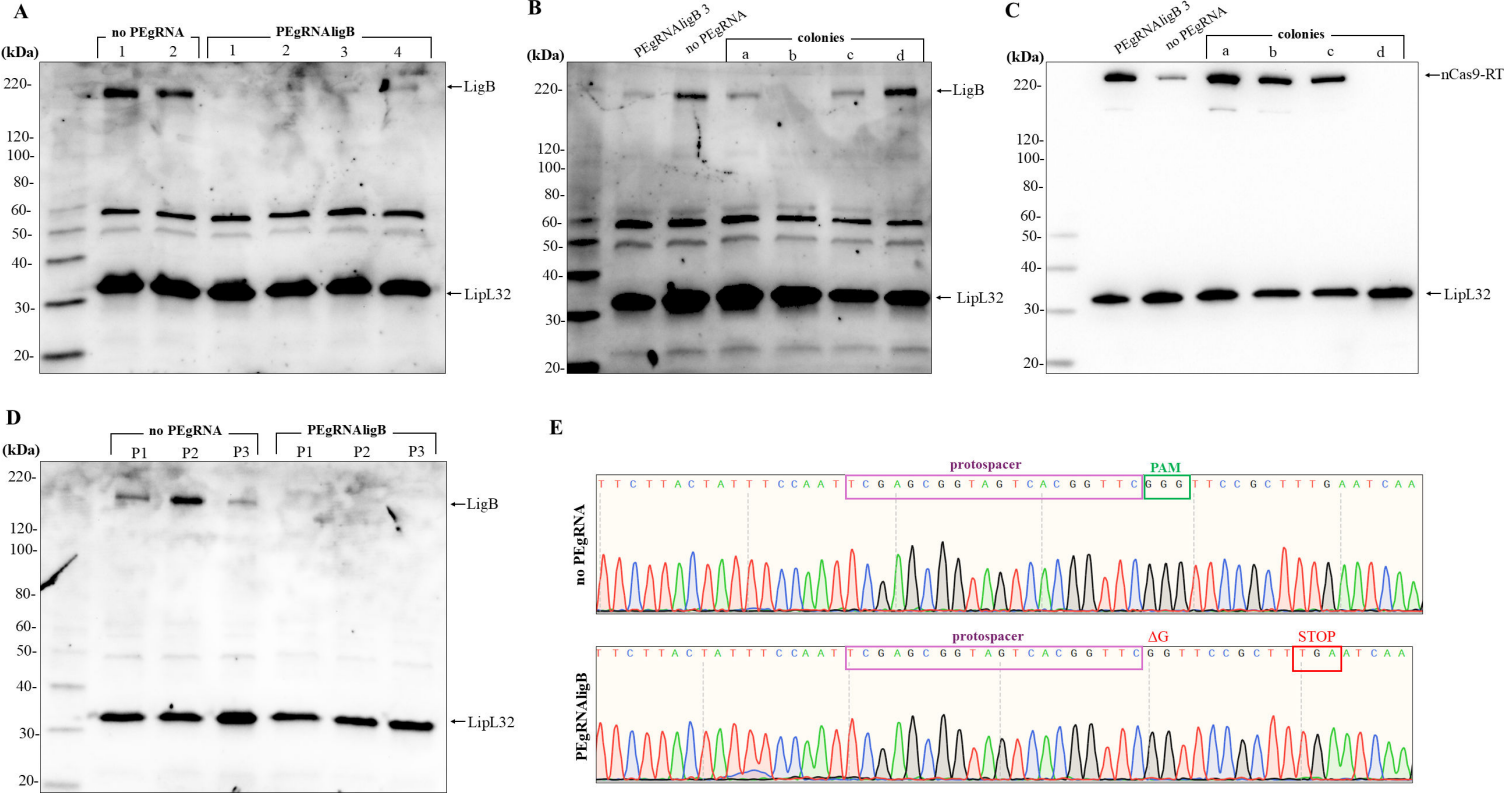
Validation of a *ligB* knockout in *L. borgpetersenii* serovar Arborea strain LR131 by CRISPR-PE. Transconjugants were selected and grown in liquid media. (**A**) Whole-cell lysates were evaluated by immunoblotting with anti-LigA/B and anti-LipL32 antibodies. Cells with an empty pMaOriPE plasmid (no PEgRNA) were used as controls. After replating of selected clones, individual colonies were re-assessed for production of LigB (**B**) and Cas9n-RT (**C**). A clone with no apparent expression of LigB and the control cells were subcultured for three *in vitro* passages (P1–P3) and re-evaluated for expression of LigB (**D**). (**E**) The *ligB* control and mutant sequences were confirmed by Sanger sequencing.

*L. borgpetersenii* colonies recovered after conjugation were randomly selected and transferred to liquid media for growth. Mid-log phase cells containing either pMaOriPE alone (control) or with the PEgRNA cassette for *ligB* (mutant) were evaluated by immunoblotting. Substantial LigB expression was observed in control cells, contrasting with faint expression of LigB that was detected in transconjugants harboring the PEgRNAligB plasmid (Fig. 1A). The diminished detection of LigB suggests a mixed population of leptospires comprising both LigB knockout and wild-type cells. To further confirm this hypothesis, the population derived from colony 3 was diluted and re-plated onto HAN plates for colony formation.

After 14 days, selected colonies were re-evaluated for expression of LigB (Fig. 1B). A mixed population was still detected in clones “a” and “c,” which showed diminished expression of LigB, while wild-type levels of LigB expression were found in clone “d.” However, no expression of LigB was detected in clone “b,” denoting a homogeneous population of knockout mutants for *ligB*. Colonies were also evaluated for the expression of the Cas9n-RT protein, which was still expressed in clones “a–c” (Fig. 1C). Because CRISPR-PE is a highly dynamic process that favors increased numbers of mutants over time, the wild-type levels of LigB in culture “d” are explained by the absence of Cas9n-RT in those cells.

Control and *ligB* knockout cells were successively subcultured *in vitro* in medium without spectinomycin and their total protein content evaluated by immunoblotting to demonstrate that their phenotypes were preserved (Fig. 1D). Sequencing of selected clones confirmed that the knockout of *ligB* was due to the PAM-disruptive one-nucleotide deletion (Fig. 1E), resulting in a premature stop codon (TGA) nine nucleotides downstream of the mutation site. No differences in growth rates for control and *ligB* KO mutants were observed (not shown).

### *L. borgpetersenii* lacking LigB can cause acute disease in the hamster model

**Fig 2:**
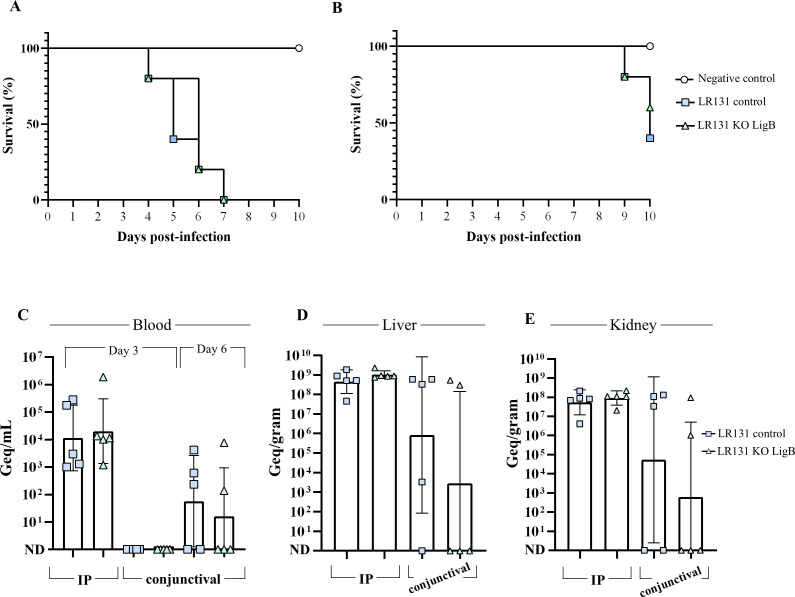
*L. borgpetersenii* lacking LigB is still virulent in the hamster model. Endpoint curves for hamsters (*n* = 5 per group) infected intraperitoneally (**A**) or by the conjunctival route (**B**) with control or *ligB* knockout LR131 cells. Animals were monitored daily for clinical signs of acute leptospirosis and humanely euthanized when endpoint criteria were met. Bacterial loads were assessed in blood on day 3 and additionally on day 6 for conjunctival infected animals (**C**), and in the liver (**D**) and kidney (**E**) at endpoint. Bacterial concentrations are expressed in genome equivalents (Geq) per mL of blood or gram of tissue.

Both control and *ligB* knockout mutants of *L. borgpetersenii* caused acute lethal disease in hamsters via the intraperitoneal (IP) (Fig. 2A) and conjunctival (Fig. 2B) infection routes. As anticipated, disease onset was delayed in animals infected via the conjunctival route (22), which more closely mimics the natural route of infection, with endpoint criteria being met on the ninth day post-infection, in contrast to the fourth day for intraperitoneal infection. Negative control animals displayed no signs of disease and were euthanized on day 10. For hamsters that received the inoculum via the conjunctival route, clinical signs of acute disease were observed in 3 of 5 control animals and 2 of 5 animals challenged with the mutant bacteria (Fig. 2B). Animals that did not display clinical symptoms were also euthanized on day 10 for comparison purposes.

The virulence of both control and *ligB* mutant leptospires was exemplified by the bacterial burden in the blood and target organs. On day 3 post-inoculation, hamsters intraperitoneally infected with control and mutant leptospires displayed a geometric mean of 11,331 and 20,164 Geq/mL of blood, respectively (Fig. 2C). For the conjunctival route, no leptospires could be detected in the blood of infected animals on day 3, in contrast to a mean of 56 and 16 Geq/mL of blood for control-infected and mutant-infected animals at day 6, respectively. Of note, no leptospires were detected in some animals inoculated via the conjunctival route, reflecting the variability associated with this route of inoculation; these animals did not display clinical signs of leptospirosis.

Following hematogenous dissemination, liver and kidney bacterial burdens were also evaluated; high bacterial loads could be detected in the liver and kidney of all animals infected by the IP route, with either control or mutant cells, when organs were harvested at indicated endpoints (Fig. 2D and E). For the liver, a mean of 4 and 9.3 × 10^8^ Geq/g were observed for control-infected and mutant-infected animals, respectively (Fig. 2D). In the kidney of these animals, control leptospires were found at 5.3 × 10^7^ Geq/g, and similarly, *ligB* mutants were found at 9 × 10^7^ Geq/g (Fig. 2E). Leptospires were cultured from liver and kidney macerates from all the intraperitoneally infected animals, and the genotypes of the liver isolates were confirmed by sequencing (Fig. S2), further corroborating that the *ligB* mutant is able to cause acute disease.

In hamsters receiving control and mutant cells via the conjunctival route of infection, leptospires were detected in the blood of those animals that later met endpoint criteria (Fig. 2C). Similarly, these same animals were the only ones in which leptospiral loads could be detected and cultured from the kidneys (Fig. 2E). No bacteria could be detected or cultured from the kidneys of the animals that did not meet endpoint criteria. For these kidney-negative animals, only one from the control group presented detectable levels of leptospiral DNA from its liver (Fig. 2D), albeit at low concentrations; however, this animal and all liver-negative animals by qPCR were also culture-negative.

### *L. borgpetersenii* lacking LigB can colonize kidneys and are excreted via urine in the rat model

Urine samples from rats infected via the IP or conjunctival routes were collected weekly from week 2 to week 6 and analyzed by dark-field microscopy and qPCR. Urine from two animals per group at week 5 was tested for culture. At the conclusion of the experiment (week 7), kidneys were aseptically removed, and organ macerates were used to inoculate liquid medium. For urinary shedding analysis and comparisons, only confirmed infected rats were considered. An animal was deemed infected if at least one detection method yielded a positive result at any time point. Based on this criterion, four and three animals were identified as infected with the control and mutant strain LR131, respectively, regardless of the infection route.

**Fig 3:**
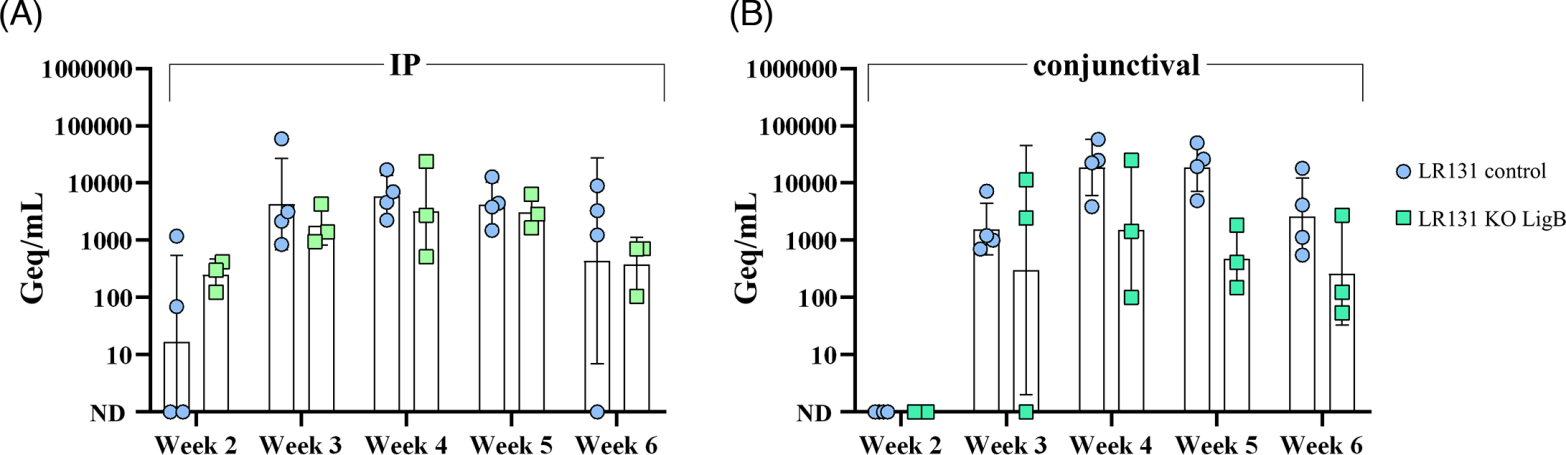
Urinary shedding of control and *ligB* mutant leptospires in the rat model of chronic leptospirosis. Outbred Sprague–Dawley rats (*n* = 4 per group) were experimentally infected with 10^7^ *L. borgpetersenii* control or *ligB* knockout cells, via the intraperitoneal (**A**) or conjunctival (**B**) route. Urine samples were collected and visualized by dark-field microscopy, and total DNA was extracted for bacterial quantification by qPCR targeting the *lipL32* gene. Numbers of leptospires in urine are expressed as genome equivalents (Geq) per mL.

Live and motile leptospires were observed in at least one animal via dark-field microscopy as early as week 2 post-inoculation in IP-infected rats from both the control and *ligB* mutant groups, consistent with qPCR detection targeting the *lipL32* gene (Fig. 3A). In contrast, and likely reflective of a delayed progression of infection via the conjunctival route, no bacteria were detected in urine by either dark-field microscopy or qPCR at this time point (Fig. 3B).

All IP-infected animals exhibited similar urinary shedding patterns across both groups, with bacterial numbers peaking at week 3, maintaining steady levels until week 5, and then declining at week 6 (Fig. 3A). Notably, in one rat infected with the control strain, leptospires were no longer detectable in urine, possibly due to renal clearance or shedding levels falling below the detection threshold. Furthermore, at week 7, no more leptospires could be detected or cultivated out of the kidney of this animal, and only one control-infected rat tested positive by qPCR. In contrast, leptospires were successfully cultured from the kidneys of all animals infected with the mutant strain.

In conjunctival infected rats, leptospires were detectable by dark-field microscopy and qPCR at week 3, with bacterial numbers comparable to the urinary shedding observed in IP-infected animals. Although a trend toward lower bacterial numbers was noted in the mutant group, the difference was not statistically significant. Leptospires were successfully recovered from the kidneys of animals infected via the conjunctival route.

Mutants recovered from urine and kidney, whether from conjunctival or IP infection routes, were subjected to *ligB* gene amplification and sequencing, confirming the preservation of the mutant genotype. Taken together, these findings demonstrate that *L. borgpetersenii* mutant *ligB* KO cells remain capable of establishing chronic infection in the rat model, regardless of the route of infection.

## DISCUSSION

The Lig proteins were first identified in pathogenic *Leptospira* by Palaniappan et al. (23) who discovered LigA through the screening of a bacteriophage library using convalescent equine sera. Subsequent studies described other members of the *lig* gene family (24), which encode LigA, LigB, and LigC—large proteins characterized by immunoglobulin-like domains. The *lig* gene family is unevenly distributed across different pathogenic *Leptospira* species and serovars and absent in saprophytic strains; *ligB* is consistently present in all pathogenic species, while *ligA* and *ligC* are only present in selected species (16), though *ligC* is a pseudogene in some strains (25). Similar proteins with immunoglobulin-like domains are recognized as virulence factors in *Escherichia coli* and *Yersinia pseudotuberculosis* (26, 27). A *ligB* knockout mutant in *L. interrogans* serovar Copenhageni strain Fiocruz L1-130 and/or *L. interrogans* serovar Manilae strain L495 retained virulence, as did knockouts for either *ligA* or *ligC* (18, 28–30). However, silencing of both *ligA* and *ligB* resulted in attenuation of virulence (19, 20).

Until recently, genetic manipulation of pathogenic *Leptospira*, and thus the identification and validation of virulence factors, was limited to a single species, *L. interrogans*. The recent advent of CRISPR-Prime Editing now allows for the assessment of virulence factors in other species (14), such as *L. borgpetersenii*, a prevalent pathogen of humans, domestic cattle, and wildlife animal species, which contains *ligB* but not *ligA*, even though some serovars can also contain an intact *ligC*. Here, we created a PAM-disruptive mutation by deleting one nucleotide from the *ligB* coding sequence, resulting in the formation of a premature stop codon and ultimately leading to the lack of LigB expression in *L. borgpetersenii* serovar Arborea strain LR131. Since this strain causes acute disease in the hamster model of leptospirosis, it provides a model to study pathogenic mechanisms in the *L. borgpetersenii* background and enables the description of conserved multispecies virulence factors that could pave the way for cross-protective subunit vaccines for leptospirosis.

The *ligB* knockout mutant was still capable of causing acute disease in hamsters following either peritoneal or conjunctival infection, similar to the control strain. No significant differences were observed in bacterial dissemination, survival curves, or bacterial loads in target organs. Furthermore, the mutant successfully colonized the kidneys of rats following either peritoneal or conjunctival infection, and mutants were recovered from their urine in numbers comparable to those of the control *Leptospira*.

Several *in vitro* studies have demonstrated overlapping properties of LigA and LigB in *L. interrogans* (16, 31–35), which was further supported by evidence showing that complete attenuation of virulence occurs only when both proteins are absent. In *L. interrogans* serovar Copenhageni strain Fiocruz L1-130, in which *ligC* is a pseudogene due to a premature stop codon, simultaneous silencing of both *ligA* and *ligB* resulted in mutants incapable of causing acute disease, although they were still able to colonize target organs (12, 20). Similarly, silencing of both *ligA* and *ligB* in *L. interrogans* serovar Manilae strain L495 also yielded avirulent mutants; however, in this case, no bacteria were recovered from target organs (19). Notably, this strain possesses an intact *ligC* gene, which suggests that this protein is not required for virulence, at least in this species background.

Several studies have highlighted the potential of Lig proteins as subunit vaccine candidates against leptospirosis (extensively reviewed by Haake and Matsunaga [16]), including formulations based on chimeric constructs (36). Moreover, experimental evidence indicates that a DNA vaccine encoding the Ig-like domains shared between LigA and LigB can mediate heterologous protection (37). A study conducted by Murray et al. (38) evaluated 238 antigens of *L. borgpetersenii* serovar Hardjo for protection against kidney colonization following homologous challenge with strain L664, which causes chronic disease in the hamster model. In this work, immunization with LigB did not protect hamsters from renal colonization, and in agreement with previous vaccine studies using recombinant LigB from *L. interrogans*, which showed that despite protection against lethal challenge, leptospires could still be recovered from target organs (39). These results contrast with a more recent study that concluded LigB was a protective vaccinogen that conferred sterile immunity against challenge in the hamster model of leptospirosis (40).

In *L. borgpetersenii*, the *ligB* gene is canonically present; in contrast, *ligA* is absent. In many cases, *ligC* is also absent, as is the case with *L. borgpetersenii* serovar Hardjo strains JB197, L550, HB203, and TC129 (6, 41–43), thus making LigB the sole representative of the Lig protein family. However, BLAST analysis of *ligC* from *L. interrogans* detected an ortholog in other serovars of *L. borgpetersenii*, including serovars Arborea, Ballum, Castellonis, and Mini. Accordingly, genome analysis of strain LR131 identified a gene that encodes an Ig-like domain-containing protein, containing 1,954 amino acids arranged in 12 Ig-like domains and an extended C-terminal region. This ortholog shares 88% similarity to LigC from *L. interrogans*.

In our *L. borgpetersenii* strain LR131 model, in which *ligA* is completely absent, the lack of LigB had no effect upon acute disease or renal carriage and urinary shedding, suggesting either that this protein is not required for virulence in this species background, or that possibly overlapping properties displayed by LigC could compensate for the lack of LigB. It is worth noting that our construct was specifically designed for *ligB* mutagenesis, with no effect on *ligC*. In future studies, the generation of a double LigB/LigC mutant in strain LR131 could help shed light on the intersecting roles of these proteins in some *L. borgpetersenii* strains; alternatively, knocking out *ligB* in serovar Hardjo, which does not have *ligA* or *ligC*, could help further elucidate its role during infection.

In conclusion, we successfully generated a knockout mutant in a virulent strain of *L. borgpetersenii*, a species that causes significant diseases in humans, domestic cattle, and wildlife. Taking advantage of the virulent phenotype for this particular strain, we can now evaluate virulence factors in a different pathogenic species. The absence of LigB did not impair virulence, but potential compensation for its loss of function by LigC in this genetic background can now be answered using the latest arsenal of tools for the genetic manipulation of pathogenic *Leptospira* spp.

## MATERIALS AND METHODS

### Bacterial strain and media

Pathogenic *L. borgpetersenii* serogroup Ballum serovar Arborea strain LR131 (8, 9) was grown in liquid HAN media (44) at 29°C under agitation. Where necessary, spectinomycin was added at 40 µg/mL. *E. coli* strain β2163 (45), auxotrophic for diaminopimelic acid (DAP), was used for general cloning and as conjugation donor cells. *E. coli* cells were grown in lysogeny broth (LB, Difco) media supplemented with DAP (0.3 mM, Sigma). Solid media were prepared by supplementing with 1.2% noble agar (Difco) or 1.5% bacteriological agar (Difco), for HAN or LB medium, respectively.

### Construction of CRISPR-PE plasmid targeting the *ligB* gene

For prime-editing guide RNA (PEgRNA) design, the *lipL32* promoter was used (46, 47), followed by a 20 nt protospacer, the Cas9 scaffold, and a 3′ extension comprising a reverse transcriptase template (RTT) sequence (13 nt, designed for a 1 nt deletion) and a 13 nt primer binding site (PBS), followed by a stretch of thymidine and the intrinsic terminator from the *bmpB* gene of *Borrelia burgdorferi* (48). The protospacer was designed based on the sequence of *ligB* from *L. borgpetersenii* strain LR131 (NCBI GenBank SRA accession SRX24593084) using CRISPRscan (https://www.crisprscan.org/). The PEgRNAligB cassette was synthesized by GeneArt (Invitrogen) and amplified by PCR with primers PEgRNA-F and PEgRNA-R (14). The resulting amplicon was used for Gibson assembly ligation with *Not*I-digested pMaOriPE (14). The final plasmid was named pMaOriPEgRNAligB. Plasmids were confirmed by sequencing with Nanopore long-read technology (Plasmidsaurus Inc., Arcadia, CA, USA) (Table S1) and were delivered to *L. borgpetersenii* by conjugation with recombinant *E. coli* β2163 cells, as per previously published protocols (13, 49).

### Electrophoresis and immunoblotting

*L. borgpetersenii* cells at mid-log to late-log phase (2–5 × 10^8^/mL) were harvested by centrifugation (10,000 × *g* for 15 min) and washed twice with PBS. Cell lysates were prepared for SDS-PAGE on 4%–15% gradient polyacrylamide gels (BioRad). For immunoblotting, proteins were electrotransferred onto polyvinylidene difluoride membranes (BioRad) by semidry transfer. Membranes were blocked with SuperBlock (PBS) Blocking Buffer (Thermo) for 1 h, and then the primary antibody diluted in the blocking buffer was added (1:20,000 for rabbit anti-LipL32, 1:5,000 for rabbit anti-LigA/B, and 1:2,000 for mouse anti-Cas9 [Sigma]). Incubation proceeded for 1 h at room temperature, and then membranes were washed three times with PBS 0.1% Tween 20 (PBS-T) and incubated with horseradish peroxidase-conjugated secondary antibodies (1:4,000) in blocking buffer. Clarity Max ECL (BioRad) was used as a chemiluminescence substrate, and blots were visualized with a ChemiDoc MP Imaging System (BioRad).

### Selection of *ligB* mutant

Transconjugant colonies were randomly selected from spectinomycin-containing agar plates and inoculated into liquid medium. Cells were harvested at mid-log phase and analyzed by immunoblotting with anti-LigA/B antibodies, and results indicated a mixture of WT and mutants. Therefore, mixed populations more likely to contain mutant cells, as indicated by decreased detection of the target protein, were selected for further enrichment by plating 100 µL at a dilution of 10^3^ cells/mL onto HAN plates without antibiotic selection. After colony formation, individual colonies were isolated and re-evaluated for the expression of LigB and Cas9n-RT. Cultures in which LigB expression was completely abolished were selected for further analysis by subculture in liquid medium without antibiotic selection. *L. borgpetersenii* cells carrying the empty pMaOriPE plasmid served as controls for wild-type LigB expression levels.

### DNA sequencing

Total DNA from knockout and control *L. borgpetersenii* cultures was used for PCR of *ligB* using primers ligB-F (5′-CATCGTTGGTTTCCATCTCCG-3′) and ligB-R (5′-TCGGCATCGAAGGAAGAACC-3′) flanking the mutation site. PCR reactions were visualized on 1% agarose gels. Amplicons were then purified, and the final product was used for Sanger DNA sequencing (50, 51) with the same primers used for amplification. Chromatograms were used for alignment with wild-type sequences by BLASTn. Amplicons were also confirmed by Nanopore sequencing (Plasmidsaurus Inc., Arcadia, CA, USA).

### Animals

Weaned female hamsters and rats were acclimated to the facility a week prior to challenge at 4–5 weeks of age. Animals were monitored daily and always had *ad libitum* access to food and water. Animals were kept in solid-bottom cages containing wood chip bedding and maintained under standard environmental conditions. To support animal welfare, environmental enrichment was provided.

### Hamster infection experiments

Outbred and recently weaned female Syrian hamsters (*Mesocricetus auratus*, *n* = 5 per group/cage, weighing 61 ± 6.8 g) were inoculated via the IP or conjunctival route with 10^7^ control or mutant leptospires. A group in which hamsters were not infected was included as a negative control. All groups were matched for weight to ensure consistency. Each animal was monitored daily, weighed for clinical signs of acute leptospirosis, and humanely euthanized upon weight loss (>10%) and/or observation of additional clinical signs (blood on paws/nose/urogenital tract, lethargy, etc.) as described previously (41). Animals were anesthetized using isoflurane and bled via retro-orbital plexus on day 3 post-infection for quantification of leptospires in the blood burden. Animals were monitored after anesthesia until full recovery, as assessed by mobility. At designated time points, hamsters were anesthetized using ketamine/xylazine and euthanized by exsanguination. One kidney and one liver lobe were harvested and immediately macerated in 5 mL of HAN media plus 5-fluorouracil (5-FU), and suspensions were used to inoculate HAN media plus 5-FU. Cultures were kept at 37°C and monitored daily by dark-field microscopy, and recovered mutants were confirmed by DNA sequencing. An additional section of the kidney and liver was harvested and immediately frozen for later analysis by quantitative PCR to measure bacterial burdens.

### Rat infection experiments and urinary shedding

Outbred female Sprague–Dawley rats (*n* = 4 per group/cage) (Envigo, Indianapolis, IN, USA), approximately 4–5 weeks old, were experimentally infected with 10^7^ *L. borgpetersenii* control or *ligB* knockout cells. Inoculation was done via intraperitoneal injection in a final volume of 0.5 mL or via the conjunctiva by applying 25 µL to the ocular and nasal mucosae for two consecutive days. To collect urine samples, rats were housed individually in metabolism cages for approximately 30 min immediately after receiving an intramuscular injection of a diuretic. Urine samples were immediately visualized under a dark-field microscope for the detection of leptospires. Additionally, 500 µL was pelleted (12,000 × *g* for 15 min), washed once with cold TE buffer, and total DNA was extracted using the Maxwell RSC PureFood Pathogen Kit (Promega, Cat# AS1660). At week 5, two animals from each cage were selected for culture of urine in HAN media supplemented with 5-FU. Infection was confirmed if at least one bacterial detection method yielded a positive result. At the conclusion of the experiment (week 7), animals were euthanized by carbon dioxide inhalation, and kidneys were harvested for bacterial culture and quantitative PCR.

### Quantification of bacterial loads

Approximately 25–75 mg of kidney cortex and liver tissue was homogenized in 500 µL of PBS using a motorized pestle. A volume corresponding to 25 mg of tissue was used for DNA extraction with the Maxwell RSC PureFood Pathogen Kit, following the manufacturer’s specifications, with a final elution volume of 100 μL.

To generate a standard curve for leptospiral genomic equivalents (Geq), genomic DNA from *L. borgpetersenii* strain LR131 was extracted, and genome size and composition were used to infer the absolute mass per genome. Bacterial load in target organs was quantified using a TaqMan-based quantitative PCR assay on a QuantStudio 3 (Thermo Fisher Scientific). The *lipL32* gene was amplified in the samples (5 μL template/well) using previously described primers (52): LipL32F (5′-AAGCATTACCGCTTGTGGTG-3′) and LipL32R (5′-GAACTCCCATTTCAGCGATT-3′). The 242 bp amplicon was detected with the LipL32-189P probe (6-carboxyfluorescein-5′-AAAGCCAGGACAAGCGCCG-3′-black hole quencher 1). PCR reactions were carried out in a total volume of 20 μL, containing 400 nM of each primer and 132.5 nM of the specific probe. The amplification protocol consisted of an initial denaturation at 95°C for 3 min, followed by 40 cycles of 95°C for 15 s and 60°C for 1 min. The concentration of leptospires, expressed as Geq per gram of tissue, was calculated based on the standard curve equation. Samples were run in triplicate and considered negative if no amplification was detected or if amplification resulted in a Geq/well below 1.

### Statistics

Bacterial loads in liver, kidney, and blood were evaluated independently by GraphPad Prism. Comparisons between control-infected and mutant-infected animals were evaluated by *t*-test. Values are expressed as geometric means and errors, and significance was determined when *P* ≤ 0.05. Endpoints of animals inoculated with mutant strains were compared with those of animals infected with control *Leptospira* cells by the log-rank test, and a *P* value below 0.05 was considered statistically significant.

## Data Availability

Plasmid sequences are deposited in GenBank under accession numbers PX766220 and PX766221.

## SUPPLEMENTAL MATERIAL

Supplemental figures iai.00662-25-s0001.pdf Fig. S1 and S2.

Table S1 iai.00662-25-s0002.docx PEgRNA cassette.

ASM does not own the copyrights to Supplemental Material that may be linked to, or accessed through, an article. The authors have granted ASM a non-exclusive, world-wide license to publish the Supplemental Material files. Please contact the corresponding author directly for reuse.
