# Supplementary material for: The Leptospira immunoglobulin-like protein LigB from Leptospira borgpetersenii serovar Arborea is not required for either acute or chronic infection
Source: Infect Immun. 2026 Mar 19;94(4):e00662-25. doi: 10.1128/iai.00662-25 (PMC13081718; doi:10.1128/iai.00662-25)
Supplement: Supplemental figures — Fig. S1 and S2. [file iai.00662-25-s0001.pdf]

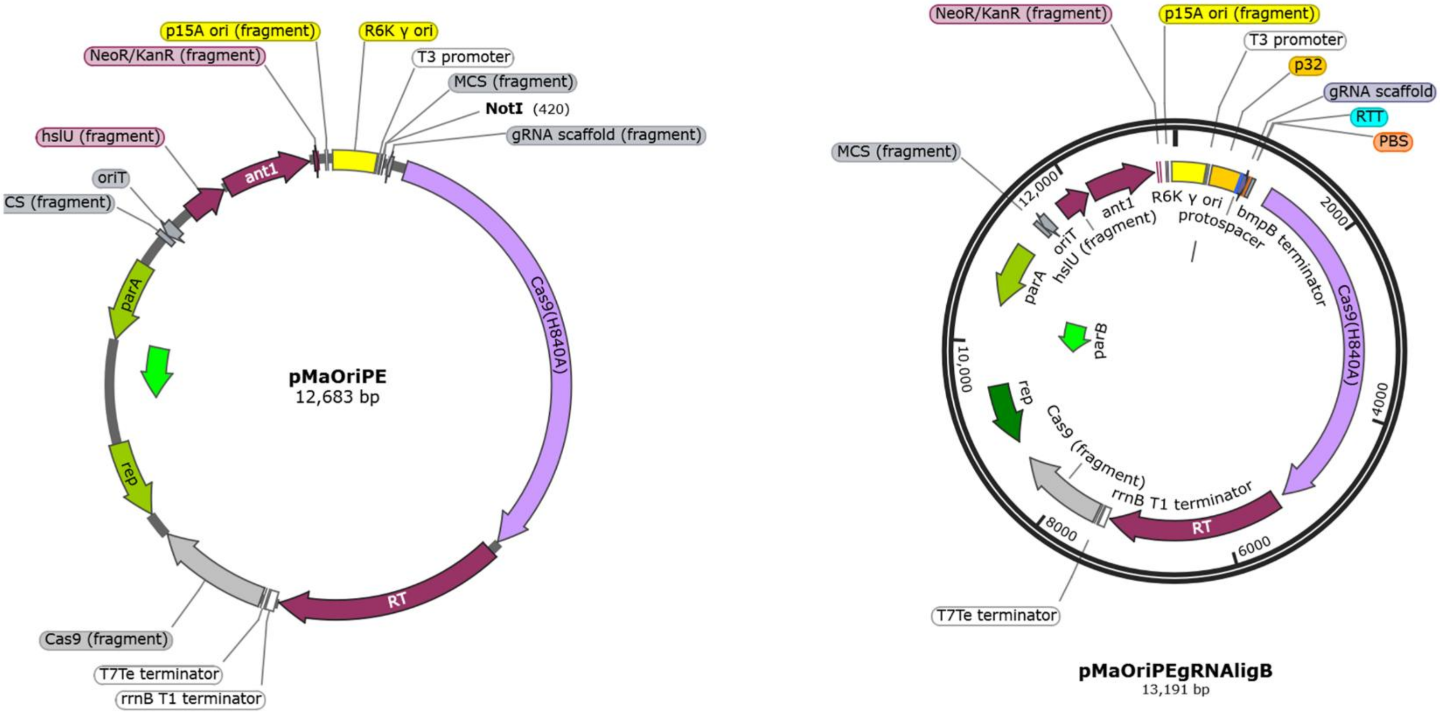

Supplementary Figure 1. Map of pMaOriPE and pMaOriPEgRNAligB. Features including the Cas9n-RT and the Cas9 fragment are displayed, along with the partition system from the pMaOri backbone, *parA*, *parB* and *rep*. The PEgRNA cassette is ligated into the *Not*I restriction site in pMaOriPEgRNAligB.

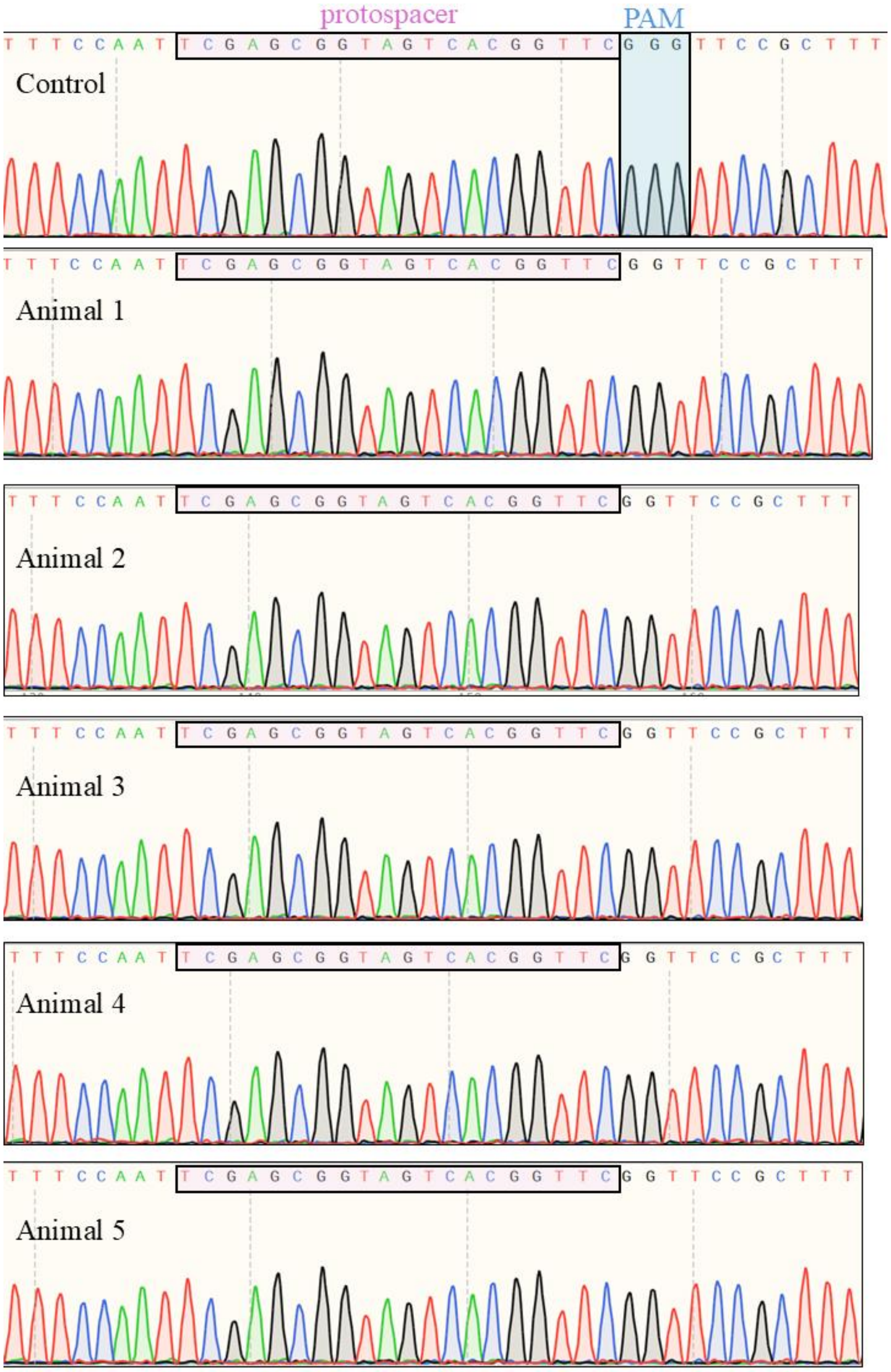

Supplementary Figure 2. *ligB* sequences from control and mutant leptospires recovered from liver. *ligB* mutants recovered from liver were used for PCR of *ligB* and sequenced by Sanger sequencing to confirm the mutation. The protospacer sequence and protospacer adjacent motif (PAM), in which the one-nucleotide mutation was created, are indicated.
