# Supplementary material for: The Leptospira immunoglobulin-like protein LigB from Leptospira borgpetersenii serovar Arborea is not required for either acute or chronic infection
Source: Infect Immun. 2026 Mar 19;94(4):e00662-25. doi: 10.1128/iai.00662-25 (PMC13081718; doi:10.1128/iai.00662-25)
Supplement: Table S1 — PEgRNA cassette. [file iai.00662-25-s0002.docx]

**PEgRNAligB cassette**

*Gcggccgc*gaacaagaaagagtcagagaattattgaagagatactcttatactaccgtctttggaagaattttcgcatggattttagatttgctggactggttgaagcgattttttcaaaaaaaataatcaatttgtgtctgagatttgaaaacgcttgtttgatagttttttaagaatttctgatgtttcaatcgtatagaaattctaaatttagaaatcatcctttacttttctctaagacttatataacaatcgctttaaactcaaattataatctttcagataaaaaattattcaatattgatttacaaaaaattcctaagttcataccgtgattttctcgagcggtagtcacggttcgttttagagctagaaatagcaagttaaaataaggctagtccgttatcaacttgaaaaagtggcaccgagtcggtgcaagcggaaccgaaccgtgactaccgctttttttcttttttggctggctatattgcagccaaaaaaagcttttcg*gcggccgc*

Promoter+protospacer+scaffold+RTT+PBS+stretch of thymidine+terminator
